# Is cellular senescence involved in cystic fibrosis?

**DOI:** 10.1186/s12931-019-0993-2

**Published:** 2019-02-14

**Authors:** Valentino Bezzerri, Francesco Piacenza, Nicole Caporelli, Marco Malavolta, Mauro Provinciali, Marco Cipolli

**Affiliations:** 1 Cystic Fibrosis Center, Azienda Ospedaliera Universitaria Ospedali Riuniti, 60121 Ancona, Italy; 2 Advanced Technology Center for Aging Research, Scientific Technological Area, IRCCS INRCA, 60121 Ancona, Italy

**Keywords:** Cystic fibrosis, Cellular senescence, SASP

## Abstract

Pulmonary disease is the main cause of the morbidity and mortality of patients affected by cystic fibrosis (CF). The lung pathology is dominated by excessive recruitment of neutrophils followed by an exaggerated inflammatory process that has also been reported to occur in the absence of apparent pathogenic infections. Airway surface dehydration and mucus accumulation are the driving forces of this process. The continuous release of reactive oxygen species and proteases by neutrophils contributes to tissue damage, which eventually leads to respiratory insufficiency. CF has been considered a paediatric problem for several decades. Nevertheless, during the last 40 years, therapeutic options for CF have been greatly improved, turning CF into a chronic disease and extending the life expectancy of patients. Unfortunately, chronic inflammatory processes, which are characterized by a substantial release of cytokines and chemokines, along with ROS and proteases, can accelerate cellular senescence, leading to further complications in adulthood. The alterations and mechanisms downstream of CFTR functional defects that can stimulate cellular senescence remain unclear. However, while there are correlative data suggesting that cellular senescence may be implicated in CF, a causal or consequential relationship between cellular senescence and CF is still far from being established. Senescence can be both beneficial and detrimental. Senescence may suppress bacterial infections and cooperate with tissue repair. Additionally, it may act as an effective anticancer mechanism. However, it may also promote a pro-inflammatory environment, thereby damaging tissues and leading to chronic age-related diseases. In this review, we present the most current knowledge on cellular senescence and contextualize its possible involvement in CF.

## Background

Cellular senescence was first described in human fibroblasts as a process that limits cell proliferation [1]. It has been well established that the senescence response observed in this pioneering study was a consequence of the progressive shortening of telomeres [2]. However, these findings are only the tip of the iceberg in terms of a complex biological response that (in addition to telomere shortening) can be triggered by a multitude of cell-intrinsic and cell-extrinsic stresses, including DNA damage, epigenetic changes, oxidative stress, chronic mitogenic signalling, oncogene activation and inactivation, and the loss of tumour suppressors [3]. Currently, cellular senescence is understood to be a state of irreversible cell cycle arrest in which cells undergo distinctive phenotypic alterations, including profound chromatin and secretome changes, and tumour suppressor pathway activation [4]. Cellular senescence cannot be characterized by a unique biomarker or phenotypic alteration because senescent cells display different alterations depending on the senescence type (e.g., replicative, DNA damage-induced or oncogene-induced), cellular origin (e.g., fibroblast or endothelial), organism (e.g., mouseor human) and temporal factors (early, full or late senescence) [4, 5]. For this reason, the characterization of cellular senescence is commonly performed by means of a combination of multiple biomarker measurements [6], including the activity of senescence-associated β-galactosidase (SAβGAL), the increased expression of cell cycle inhibitors (p16, p53, and p21), the presence of DNA damage foci containing activated γH2AX (a marker of the DNA damage response), the decondensation of peri-centromeric satellite DNA (a phenomenon termed senescence-associated distension of satellite, SADS), a lack of markers of proliferation (Ki67 expression or BrdU incorporation), the loss and relocalization of the nuclear protein high mobility group box 1 (HMGB1), staining for lipofuscins, and morphological changes (enlarged and irregular cells). Cellular senescence is thus a state of the cell that is clearly different from quiescence, which is defined as a “reversible non-proliferating state” that is due to cell cycle arrest [3, 7]. However, cellular quiescence and senescence share several common signalling pathways. For instance, SAβGAL reactivity is observed in both senescence and quiescence even though reactivity is clearly higher in senescence [8]. Additionally, the tumour suppressor p53 plays a critical role in both reversible quiescence and irreversible senescence [9]. Maximal activation of p53 leads to quiescence, whereas its partial activation leads to senescence under certain conditions [10]. The mammalian target of rapamycin (mTOR) serine/threonine protein kinase can instead force the cell fate towards senescence over quiescence [8, 11, 12]. Moreover, these two processes may present distinctive molecular mechanisms that underlie cell cycle arrest. In fact, during senescence, the cell cycle is permanently blocked by p53-dependent p21 activation or by p16 [13], whereas during quiescence, cell cycle arrest is mostly mediated by the CDK inhibitor p27 [9, 14]. Another distinctive feature of most senescent cells is the development of a secretory phenotype, termed the senescence-associated secretory phenotype (SASP), which includes several proteins involved in inflammation processes, proteases, and haemostatic and growth factors [15].

Senescence can be both beneficial and detrimental. On the one hand, as mentioned above, senescence acts as an effective anticancer mechanism by preventing malignant transformation and by limiting tumour progression. Moreover, acute senescence can be beneficial during embryonic development, wound healing and tissue repair [16]. However, excessive accumulation of senescent cells has been demonstrated in a multitude of aged tissues. Increased resistance to apoptosis, decreased senescence immunosurveillance, an increased rate of senescence-inducing damage and “bystander effects” (a phenomenon in which senescent cells propagate the senescence of neighbouring cells through the SASP or through other uncharacterized mechanisms) have been proposed as mechanisms to explain why there is a consistent accumulation of senescent cells in ageing and in age-related diseases [17]. Excessive accumulation of senescent cells may thus promote a pro-inflammatory environment mediated by the SASP, which contributes to reduced tissue regeneration and organ dysfunction [4, 18]. In fact, senescent cells that persist for a prolonged time within tissues lead to deterioration processes and chronic age-related diseases [16]. This phenomenon is related to the inflammatory status promoted by the senescent cells combined with the exhaustion of the stem cell pools and the resulting impairment in regenerative capacity. Indeed, the accumulation of senescent cells was recently observed in a transgenic mouse model (Sox2−TK mice) in which adult SOX2^+^ stem cells were depleted by the administration of ganciclovir [19]. Nevertheless, cellular senescence may also contribute to stem cell exhaustion through the SASP and the bystander effect [20], thus suggesting the existence of a vicious circle that contributes to tissue degeneration.

Importantly, lung aging in mice has been reported to be associated with higher levels of IL-6 and transforming growth factor beta (TGF-β) expression and the increased expression of the senescence-inducing cyclin-dependent kinase inhibitors (CDKi) p16 and p21 both in airway epithelia [21] and in vascular smooth muscle cells [22]. Senescent cells have been observed in several pathologies, including idiopathic pulmonary fibrosis (IPF) and chronic obstructive pulmonary disease (COPD) [16]. The lungs of COPD patients resemble those of subjects exhibiting normal lung aging, namely, increased expression of senescence-associated soluble markers and increased expression of p16 and p21, as reported in airway epithelial cells and in vascular endothelial cells [21]. Similarly, several biomarkers of cell senescence, such as increased p21 expression and SAβGAL activity, have been found in IPF alveolar, bronchial and mesenchymal cells both in humans and in mice [23]. Of note, a senescence-like phenotype has also been proposed in cystic fibrosis (CF). In fact, primary bronchial epithelial cells obtained from 9 patients with CF showed increased levels of p16 and DNA damage response markers, such as phospho-histone 2AX and phospho-checkpoint kinase 2 [23]. In the same study, the authors showed that neutrophil elastase (NE) was able to induce p16 accumulation in primary normal bronchial epithelial cells in a dose-dependent manner [23]. Moreover, neutrophils obtained from the bronchoalveolar lavage fluid (BALF) of patients with CF expressed p21 [24], which has been reported to modulate the delayed neutrophil apoptosis observed in CF [25]. Airborne particulate matter increased p21 expression in CF bronchial epithelial cells through mitochondrial stress activation [26]. Although the presence of senescent cells in CF airways is far from firmly established and although data regarding this point are only correlative, these results suggest that the excessive NE release observed in CF lungs might accelerate the senescence process in CF.

CF is an inherited disease caused by mutations in the cystic fibrosis transmembrane conductance regulator (*CFTR*) gene. *CFTR* encodes a chloride channel that is widely expressed in human epithelia [27]. Mutations affecting *CFTR* expression or function lead to defective chloride efflux followed by sodium absorption by the amiloride-sensitive epithelial Na + channels (ENaC). This process underlies dehydration, particularly within the bronchial lumina of CF patients. Dehydration of airway surface liquid impairs mucociliary clearance, favouring inflammation processes that are dominated by neutrophil infiltrate [28]. CF patients present chronic lung inflammation, which has been observed in young subjects and animal models in the absence of apparent bacterial infections [29]. After bacterial infections, mainly sustained by *Pseudomonas aeruginosa*, inflammation is amplified, leading to the exaggerated recruitment of neutrophils within the bronchial lumen, which unfortunately produces an ineffective antibacterial response [30]. The inflammatory process is mainly driven by the activation of the nuclear factor (NF)-κB [31], which in turn leads to the overexpression of the neutrophil chemoattractant interleukin (IL)-8 (CXCL-8) [32].

In normal subjects, lung maturity peaks between 20 and 25 years of age. After this period, lung functions gradually decline in terms of the forced expiratory volume in 1 s (FEV1) and forced vital capacity (FVC), ultimately leading to respiratory insufficiency over time [16]. Similar to CF conditions, ageing has been reported to modify lung structure by decreasing mucociliary clearance, reducing the inner surface area for exchanging gas and causing a constitutive pro-inflammatory status. This process is mainly mediated by reactive oxygen species (ROS) and proteases, in particular NE, that are released by innate immune cells. The accumulation of ROS leads to mitochondrial oxidative damage, decreasing the mitochondrial copy number in animal models [33]. Increased levels of ROS are associated with the augmented activity of the tumour suppressor protein p53 and subsequent upregulation of p21 and p16 protein expression both in vitro and in vivo [34, 35]. By means of BALF obtained from healthy individuals from discontinuous age groups, neutrophil recruitment was demonstrated to increase with age [36]. This process promotes the continuous release of NE, which has been shown to degrade elastin, impairing pulmonary elastic recoil. The basal pro-inflammatory status due to continuous oxidative stress signalling fosters the release of cytokines and chemokines, particularly IL-6 and IL-8 [36]. Similarly, CF is characterized by the recruitment of a large number of neutrophils, which leads to the sequential, ROS- and protease-driven degradation of lung parenchyma [37]. The appearance is that of CF somehow accelerating the physiological ageing process, leading to a prematurely aged microenvironment that further exacerbates the decline in lung function.

Ageing and senescence in chronic inflammatory diseases are becoming increasingly recognized by the scientific community. Because the life expectancy of CF patients is considerably increasing due to novel therapeutic options, further study is necessary to investigate whether the senescence process plays a role in the chronic inflammation of CF.

## Mitochondrial dysfunction is a common feature of senescence and CF

Mitochondria are major players in cellular redox homeostasis. Mitochondria can transduce a multitude of cell signals from various pathways, and these signals mainly converge on the production of ATP and ROS. Increased ROS production has been associated with mitochondrial oxidative damage and a reduction in mitochondrial copy number [33, 38–40]. Moreover, ROS production increases with ageing. As mentioned above, stem cell exhaustion is a characteristic feature of ageing [18]. In chronic lung diseases, such as chronic obstructive pulmonary disease (COPD), stem cell self-renewal is slower than the rate of differentiation due to continued tissue damage and subsequent regeneration. This leads to a decline in the cell population as a result of exhaustion. ROS play key roles in this process, as they regulate cell proliferation, force out quiescent cells, and inhibit the FOXO-dependent stress response and autophagy [18]. Because senescence-mediated stem cell exhaustion has not yet been investigated, a comprehensive analysis of mTOR- and ROS-dependent senescence signalling deserves particular attention. These studies might reveal similarities or differences with COPD that would be useful for understanding the importance of the senescence process in CF. Senescent rats show increased accumulation of mitochondrial oxidation products, such as thiobarbituric acid-reactive substances and protein carbonyl, which progressively impair mitochondrial functions [41]. Oxidative stress in the kidneys of mice consuming a high-fat diet show increased generation of mitochondrial oxidants through a member of the NADPH oxidase family, namely, Nox4 [42]. Nox4 has also been reported to act as the driving force for superoxide production in the cardiovascular system. Increased Nox4 activation has been associated with increased mitochondrial ROS release, mitochondrial dysfunction, and myocardial cell death during cardiac hypertrophy [43]. Interestingly, Nox4 gene expression has been shown to increase with age in different animal models [44, 45]. Mitochondrial ROS release leads to the formation of the inflammasome, a large protein complex. The inflammasome family consists of four subfamilies, namely, NLRP3, NLRP1, NLRC4, and AIM2 [46–48]. Among these complexes, NLRP3 has been the most characterized [49] and has been proposed as a sensor of mitochondrial status. After activation, inflammasomes generally trigger the proteolytic maturation of pro-inflammatory cytokine precursors, such as IL-1β [48]. That the serine-threonine kinasemTOR can regulate the SASP in a mitochondria-dependent manner has been suggested [50]. mTOR-induced SASP translation leads to the increased release of IL-1α, which in turn enhances NF-κB phosphorylation and its nuclear translocation, ultimately increasing the senescence process [51]. Notably, mTOR activity has been found to be upregulated in CF bronchial epithelial cells, and inhibition of mTOR signalling pathways shows increased CFTR stability and expression [52]. Depletion of mitochondrial DNA (mtDNA), knockdown of mitochondrial sirtuin-3 (SIRT3) or inhibition of the electron transport chain can induce mitochondrial dysfunction-associated senescence [53]. Dysfunctional mitochondria release multiple damage-associated molecular patterns (DAMPs), such as ATP, ROS and mtDNA, activating the NLRP3 inflammasome, which in turn increases the expression of IL-1 and leads to the activation of the NF-κB pathway, supporting senescence biogenesis [54]. In addition to its function as a chloride channel, CFTR indirectly regulates the expression of several other genes, including CISD1 [55] and MT-ND4 [56]. These two genes encode mitochondrial proteins whose gene expression levels are reduced in CF cells [57]. In particular, MT-ND4 encodes the ND4 protein, which is an important subunit of mitochondrial Complex I (mtCx-I) that modulates its activity [58]. mtCx-I activity is indeed downregulated in CF cells [57, 59–62]. Furthermore, mitochondrial fragmentation and decreased mitochondrial membrane potential due to changes in mitochondrial Ca^2+^ homeostasis have been reported in CF cells [63]. Thus, the activities of mtCx-I and mtCx-IV are impaired in CF conditions, while mitochondrial ROS production and membrane lipid peroxidation are increased [64]. Mitochondrial dysfunction has recently been reported to lead to NLRP3 inflammasome activation in CF, contributing to *P.aeruginosa*-dependent inflammation in bronchial epithelia. This process underlies the inflammasome-dependent release of IL-1β and IL-18 [65]. Interestingly, IL-1β blocking antibody or mitogen-activated protein kinase (MAPK) p38 inhibitor SB203580 can significantly decrease the mitochondrial ROS level to near baseline values in CF cells [66]. Notably, the FDA-approved small molecule VX-809 (lumacaftor) [67, 68] and the preclinically tested molecule 4,6,4′-trimethylangelicin [69], which act as correctors of the F508del-mutated CFTR in vitro, partially restore CF-dependent mitochondrial failure [64]. Taken together, these findings suggest that mitochondrial dysfunction-associated cellular senescence might be involved in CF and that the correction of CFTR function can also ameliorate senescence signalling. Nevertheless, further study of mitochondrial-induced senescence in CF cells may unravel new molecular targets to develop anti-inflammatory therapies.

## Pro-inflammatory signal transduction: Similarities between SASP and CF

Senescence is characterized by cell cycle arrest, which is blocked at S-phase entry through the activation of different signalling pathways, such as the tumour suppressor protein p53 pathway, which in turn leads to the upregulation of p21. Alternatively, senescence can occur via the p16 and p14 pathways or via crosstalk between the p53-p21 and p16 pathways [70]. Although the cell cycle is arrested during senescence, cell metabolism remains active. However, protein expression and secretion patterns significantly change, ultimately leading to a the SASP [15]. The SASP consists of the release of several families of soluble (interleukins, chemokines, growth factors and proteases) and insoluble (extracellular matrix components) factors that can activate different cell-surface receptors, triggering a multitude of signal transduction pathways that in turn may promote inflammation and cancer [15].The SASP has also been proposed as a mechanism for resolving stress-induced senescence through the release of immunomodulator molecules that are able to trigger the clearance of senescent cells by phagocytosis [71]. SASP production and the resulting components depend on the cell type and senescence mechanism. However, IL-6, which induces paracrine stress-induced senescence in mouse and human keratinocytes, melanocytes, monocytes, fibroblasts, and epithelial cells, has been frequently described as the main pro-inflammatory cytokine released by the SASP [15]. Most senescent cells overexpress IL-8, along with growth regulated oncogene (GRO)α and GROβ [15]. Importantly, IL-6, IL-8 and GRO isoforms are also hallmarks of CF inflammation [31]. The ageing process and senescence have been suggested to be accelerated under chronic inflammatory processes. In vivo studies have revealed that NF-κB plays a key role in the inflammation-dependent acceleration of senescence in the guts and livers of mice [72]. Moreover, the nuclear NF-κB levels increase with age both in mice and in rats [73]. SASP is initiated by NF-κB signalling through the activation of p38 and is subsequently maintained by IL-1α in an autocrine manner [74, 75]. In this context, NF-κB also cooperates with the CCAAT/enhancer-binding protein (CEBP)/β transcription factor during the senescence-mediated release of IL-6, IL-8, TGF-β and proteases [71]. Similarly, NF-κB is recognized as a key regulator of IL-8via its cooperation with C/EBPβ expression in CF cells [32, 76]. Notably, TGF-β levels are upregulated in the plasma and BALF of paediatric CF patients [77]. After *P.aeruginosa* infection, TGF-β release is further increased, contributing to the paracrine induction of lung fibrosis in CF. Paracrine activation of the TGF-β pathway plays an important role in inducing ROS release [71]. ROS trigger the activation of the MAPK cascade through the MEK and ERK signalling pathways, which in turn activate p38, and this process has been shown to regulate p53-dependent upregulation of p21 expression [78]. Inflammation and oxidative stress play key roles in the senescence of immune cells, regulating gene expression and the release of several factors in the bone marrow, including IFN-y, TNFα, IL-15 and IL-6 [79, 80]. Treatment with antioxidants, including N-acetyl cysteine (NAC) and vitamin C, reduces cytokine release in bone marrow, thus suggesting that antioxidant therapy may be beneficial in counteracting immunosenescence [79, 80]. Interestingly, CF cells present increased ROS levels, which have been proposed to promote defective autophagy [81]. Autophagy is acatabolic pathway that deteriorates intracellular proteins and organelles through the lysosome [82, 83]. Notably, defective autophagy increases susceptibility to ROS signalling and apoptosis, whereas activation of autophagy leads to inhibition of apoptosis [84]. Over time, damaged and misfolded proteins accumulate into the cells through a functional impairment in autophagy, thus contributing to cellular senescence. Autophagy and cellular senescence are stress responses that regulate homeostasis. Additionally, the SASP may preserve tissue homeostasis by increasing immune surveillance of damaged cells. Through molecular mechanisms that involve mTOR, autophagy promotes a high rate of recycling of amino acids and other metabolites, which are subsequently used by the mTORC1 complex to synthesize SASP factors, thus facilitating senescence. Conversely, autophagy inhibition has also been shown to induce cellular senescence in normal proliferating cells [85]. In this regard, the senescence regulator GATA4 has been suggested to tip the scales in favour of autophagy-driven senescence rather than homeostasis [85].

*P.aeruginosa*-dependent IL-8 expression in bronchial epithelial cells has been previously reported to be mainly driven by NF-κB activation through the MEK-ERK and p38 signalling cascade [32]. The specific inhibitor of p38, namely, SB203580, can indeed reduce CF-related IL-8 overexpression [32, 86]. Interestingly, the same inhibitor can prevent sarcopenia (in vitro and in vivo) [87, 88], an age-related syndrome characterized by the loss of skeletal muscle mass and function that is tightly associated with the cellular senescence of muscle stem cells.

Loss of caveolin (Cav)-1 expression is protective against bleomycin-induced lung fibrosis with reduced SASP release in a mouse model of IPF, suggesting Cav-1 as an important player during the SASP process and during senescence [89]. Notably, the loss of CFTR expression in primary human alveolar macrophages is accompanied by increased Cav-1 expression at both the mRNA and protein levels. Increased Cav-1 expression is associated with increased NF-κB nuclear translocation and subsequent augmentation of IL-8 expression in CFTR-deficient cells [90].

Taken together, these findings suggest that CF conditions may lead to an accelerated SASP process. Loss of CFTR activity leads to increased NF-κB and C/EBPβ activity through MAPK p38 signalling, ultimately promoting the expression of SASP factors IL-6, IL-8, GROα, GROβ and TGF-β [31, 32, 76, 77, 86, 91–93].

## Cellular senescence is associated with the high risk of cancer observed in CF

Over the last 30 years, the long-term survival rate of CF patients has greatly improved. Unfortunately, an elevated risk of cancer development has been associated with survival in CF (Table 1).

**Table 1:** Risk of cancer associated with CF and the role of CFTR in oncogenesis

Organ	Cancer type	Role of CFTR	Ref
Airways	Nasopharyngeal carcinoma	Low expression of CFTR is associated with advanced stage, distant metastasis and poor prognosis. Overexpression of CFTR inhibits cell migration and tissue invasion.	[136]
	Non-small cell lung cancer	Loss of CFTR expression is observed in NSCLC cells. Increased methylation of the CFTR gene is associated with poor prognosis in young patients.	[100]
	Non-small cell lung cancer	Reduced CFTR expression is associated with advanced stage and metastasis. CFTR overexpression leads to the suppression of cancer progression in vitro and in vivo.	[101]
	Lung squamous cell carcinoma	Reduced CFTR expression increases metastasis	[137]
	Other types of lung cancer	The F508del mutation plays a protective role in terms of lung cancer risk.	[138]
Intestine	Colorectal cancer	CFTR knockout enhances malignancy in mice, due to activation of the ERK-1/2 pathway and a reduction in epithelial tightness.	[139]
	Colorectal cancer	Loss of CFTR expression leads to tumour development in the mouse gut. Reduced CFTR expression is associated with a poor prognosis for CRC patients.	[104]

A high incidence of gastrointestinal cancer has been observed in CF patients. The colorectal cancer (CRC) risk was found to be 24-fold elevated in CF patients [94]. Increased levels of IL-6, IL-8 and IP-10 in serum have been associated with poor CRC prognosis and liver metastasis. In particular, IL-8 contributes to cell proliferation and the migration of malignant cells and is involved in angiogenesis both in vitro and in vivo [95, 96]. Because the SASP induces the release of IL-8 and IL-6, which are already upregulated in CF, cell senescence might contribute to enhancing the redundant activation of these pro-tumoural cytokines. In this context, it should be considered that senescent cells can be eliminated by the immune system through a mechanism that involves the SASP. This type of response is named senescence immune surveillance, and the failure of this mechanism has been shown to promote oncogenesis and tumour growth in different models [97, 98]. Interestingly, monocyte/macrophage dysfunctions, including defects in phagocytosis, have been identified in CF [99]. These defects may contribute to the excessive accumulation of senescent cells, which in turn may promote a pro-tumourigenic microenvironment.

Moreover, DNA methylation and mutations of CFTR have been identified in non-small cell lung cancer (NSCLC), which is a common lung malignancy characterized by poor long-term survival that is mainly due to metastasis and tumour relapse [100, 101]. Epigenetic patterns change over the organismal lifespan, indicating that epigenetic variants play an important role in ageing. Higher levels of DNA methylation at a promoter-associated CpG island primarily lead to reduced gene expression, whereas hypermethylation across the entire gene is generally associated with increased gene expression [102]. However, gradual changes in DNA methylation occur with age [102]. Of note, hypermethylation of CFTR or the F508del CFTR has been suggested as a tumour risk biomarker for young CF patients [100, 103].

In an analysis of 296 patients affected by NSCLC, reduced CFTR expression was observed to be significantly associated with an NSCLC propensity, NSCLC metastasis and poor prognosis [101]. Interestingly, CFTR has also recently been proposed as a tumour suppressor protein [104]. The authors observed that the specific knockout of CFTR expression in the mouse gut leads to a significantly increased incidence of tumour development in the colon and in the entire small intestine. A higher risk of tumour progression seems to be partially due to altered Wnt/β-catenin-dependent gene expression [104]. However, a recent single-cell study reported different levels of CFTR expression, depending on the cell type both in humans and in mice [105]. The authors identified a novel population, termed ionocytes, and although they make up only 1–2% of epithelial cells, ionocytes seem to be the main source of CFTR activity in the lung epithelium. These findings raise additional questions regarding CFTR-dependent carcinogenesis that deserve more thorough investigations.

Deregulated apoptosis is a hallmark of cancer. During the senescence process, the deregulation of apoptosis varies depending on the tissue type, but there is substantial evidence that resistance to apoptosis contributes to the accumulation of senescent cells in ageing and in several age-related diseases, including COPD and IPF [106, 107]. Whether a similar phenomenon can be part of the CF phenotype remains to be established. In contrast, there are several studies supporting a role for CFTR mutations in promoting the apoptosis of normally dividing cells.

The most common mutation reported in CF is F508del*CFTR*, which causes misfolding of the CFTR protein and its retention in the endoplasmic reticulum (ER) [108]. Protein misfolding triggers a mechanism known as the unfolded protein response (UPR), involving ER stress and NF-κB activation [109]. The UPR leads to a reduction in protein synthesis and the release of cytokines, which in turn induce apoptosis [110]. Additionally, ceramide accumulation in CF airways has been reported to induce lung inflammation and the subsequent apoptosis of epithelial cells with concomitant deposition of DNA in the airways, which increases the adhesion of opportunistic bacteria [111–114].

Taken together, these findings highlight the need for focused investigations of senescence signalling in CF.

## Conclusions

Since the first report of CF that was published in 1938, in which Dorothy Andersen investigated 49 children showing a pathology characterized by exocrine pancreatic insufficiency associated with lung disease [115], the survival of patients with CF has risen from a few months to over 40 years [116]. The increased life expectancy is now promoted further by a new class of drugs termed CFTR modulators.

Over the last decade, CFTR modulator science has greatly advanced. Currently, the number of CF patients who are candidates for CFTR modulator therapy is higher than 50% and may reach up to 90% within a few years [117]. Ivacaftor (VX-770) was the first effective CFTR modulator to draw attention to this new class of molecules [118, 119]. Ivacaftor corrects gating-defective mutations of CFTR, such as the G551D variant, which is found in approximately 4–5% of CF patients. The major evidence for ivacaftor was reported in terms of clinical benefit: the drug significantly improved the percent predicted FEV1 by more than 10% and reduced the risk of pulmonary exacerbations by more than 50% [120]. Lumacaftor (VX-809) is a corrector molecule that is able to facilitate the trafficking and plasma membrane localization of the F508del CFTR [68, 121]. The F508del is the most common CFTR mutation. Among Caucasian CF patients worldwide, the frequency of F508del mutation varies from a minimum of 20% in Turkey to a maximum of 100% in the Faroe Islands of Denmark [108]. In US, its frequency is almost 72% in Caucasian population, 31–44% in African Americans, and 18% in Iranians [122–124]. Although both in vitro and in vivo studies have proven that lumacaftor can improve the maturation and function of the F508del CFTR protein [68, 121], lumacaftor monotherapy has shown insufficient clinical benefits in CF adults who are homozygous for the F508del CFTR, despite a moderate but significant reduction in sweat chloride [67]. A lumacaftor derivative, termed tezacaftor, in combination with ivacaftor, led to a modest improvement in the FEV1, a reduction in the pulmonary exacerbation risk and an improvement in weight in a cohort of CF subjects who were homozygous for the F508del CFTR [125, 126]. Furthermore, there are currently several new CFTR corrector and potentiator molecules in clinical trials, and these studies are expected to provide further insights into increasing CFTR expression and function in patients [127].

**Fig. 1:**
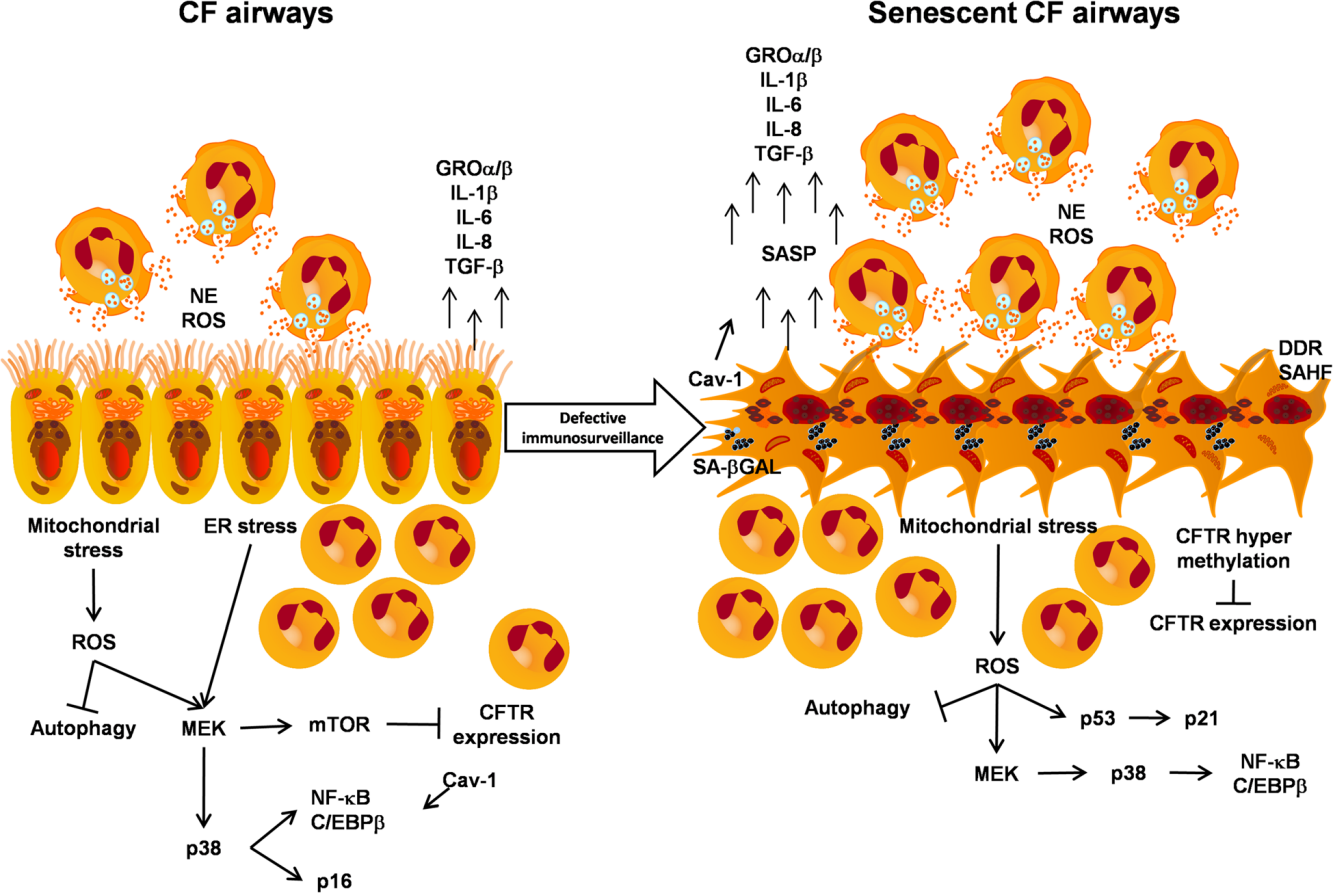
Hypothesis of cellular senescence in CF airways. Signal transduction pathways are commonly activated in CF epithelial cells (left) and are hypothesized to be involved in senescent CF cells (right) upon defective senescence immunosurveillance. CF senescent cells might promote further recruitment of neutrophils (PMNs) into the bronchial lumen through the activation of ROS and mitochondrial stress signalling, which in turn increase SASP release, worsening the lung inflammatory process and activating pro-tumoural pathways. DDR, DNA damage response; SAHF, senescence-associated heterochromatin foci

Unfortunately, these drugs will not be able to regenerate exhausted lung tissues, and whether the release of SASP and senescence signalling reported in CF lung epithelia can be affected by CFTR modulators is unclear. However, the extended life expectancy of CF subjects can promote senescence, which is early activated in patients with CF. Thus, in addition to the correction of CFTR dysfunction, the new challenge in treating older patients might be to control the accelerated aging processes, which are associated with inflammation, tissue damage and cancer development. While there are consistent data to support the hypothesis that cellular senescence may be implicated in CF (Table 2), the alterations and mechanisms downstream of the CFTR functional defects that can stimulate senescence in the lung and in other organs affected by the disease remain unknown. Most importantly, the role of cellular senescence in CF has not been investigated thus far. It is expected that the inflammation driven by an excess of SASP-producing cells may play a deleterious role in the progression of the disease (Fig. 1). However, we cannot exclude the possibility that senescent cells may play a beneficial role, suppressing bacterial infections and cooperating with tissue repair. This gap in knowledge is clearly a limit for testing bioactive compounds that have already been proven to have senolytic activity in clinical settings [3, 128–130]. The recent discovery that azithromycin exhibits senolytic activity in human fibroblasts [131] further supports our hypothesis to target senescent cells in CF. Indeed, azithromycin is an antibiotic used to treat patients with CF and is also known to have an anti-inflammatory effects [132, 133]. The evidence of a senolytic activity by azithromycin suggests that the anti-inflammatory effects may be the consequence of the selective removal of senescent lung fibroblasts and the subsequent decline in the production of SASP. To fill the gap and unravel the mechanisms involved, it would be useful to study the effects of senescent cell removal in mouse models of CF engineered with a transgene that allows the selective removal and visualization of senescent cells (e.g., by breeding with p16-3MR mice). This strategy has been successfully used to demonstrate the deleterious role of cellular senescence in atherosclerotic [134] and osteoarthritis [135] mouse models, and it is likely that this will be the key to understanding the role of cellular senescence in CF.

**Table 2:** Senescence-related processes reported in CF

Senescence-related process	Comparison with CF condition	Ref
Increased PMN recruitment into the bronchial lumen with ageing	Increased PMN recruitment within the bronchial lumen associated with increased release of chemokines	[23, 30, 36]
SASP release	Increased levels of IL-6, IL-8, IL-1β GROα and TGF-β	[15, 32, 65, 77, 91]
Dysregulated apoptosis: SASP and defective autophagy induce apoptosis, whereas ageing reduces apoptosis, promotes carcinogenesis and reduces immunosurveillance.	Increased apoptosis mediated by cytokines and ceramide accumulation in lung epithelia and a p21-dependent decrease in the apoptotic rate of PMNs.	[25, 106, 111, 112]
Increased NE release with ageing due to accumulation of PMNs	Early increase in NE accumulation into the bronchial lumen due to excessive accumulation of PMNs	[23, 30, 36]
Mitochondrial stress	Increased ROS levels and ATP release due to mitochondrial impairment	[48, 57, 60, 62]
Inflammasome activation	NRLP3-mediated inflammasome activation and increased IL-1β release	[36, 48, 53, 65]
mTOR-dependent increase in SASP with subsequent upregulation of the NF-κB pathway	Upregulated mTOR activity is linked to decreased CFTR stability and expression.	[8, 10, 12, 14, 52]
Increased p21 activation mediated by upregulation of the p53 pathway	Upregulation of the p21 pathway in PMNs and bronchial epithelial cells, mediated by mitochondrial stress signalling.	[23, 24, 26, 140]
Increased p38 MAPK signalling transduction	p38 pathway upregulation leading to NF-κB activation in bronchial epithelia	[32, 75, 86]
NF-κB and C/EBPβ increased activation	Increased NF-κB and C/EBPβ nuclear translocation associated with increased cytokine expression in bronchial epithelia	[32, 73, 75, 86]
Cav-1 involvement in SASP	Loss of CFTR expression leads to Cav-1 upregulation and a subsequent increase in cytokine release and NF-κB activation.	[89, 90]

## Abbreviations

Cav-1: Caveolin-1
CDKi: Cyclin-dependent kinase inhibitors
CEBP/β: CCAAT/enhancer-binding protein
CF: Cystic fibrosis
CFTR: Cystic fibrosis transmembrane conductance regulator
COPD: Chronic obstructive pulmonary disease
CRC: Colorectal cancer
DAMP: Damage-associated molecular pattern
FEV1: Forced expiratory volume in 1 s
FVC: Forced vital capacity
GROα: Growth regulated oncogene alpha
GROβ: Growth regulated oncogene beta
IL-6: Interleukin-6
IL-8: Interleukin-8
IPF: Idiopathic pulmonary fibrosis
MAPK: Mitogen-activated protein kinase
mTOR: Mammalian target of rapamycin
NF-κB: Nuclear factor κB
NSCLC: Non-small cell lung cancer
ROS: Reactive oxygen species
SASP: Senescence-associated secretory phenotype
SAβGAL: Senescence-associated beta galactosidase
SIRT3: Sirtuin-3
TGF-β: Transforming growth factor beta
